# High ^18^F-fluorodeoxyglucose uptake in primary bilateral adrenal diffuse large B-cell lymphomas with nongerminal center B-cell phenotype: A case report

**DOI:** 10.1097/MD.0000000000010480

**Published:** 2018-04-27

**Authors:** Jing Zhou, Yigang Zhao, Zhengxing Gou

**Affiliations:** Department of Nuclear Medicine, Fuling Central Hospital of Chongqing, Chongqing, China.

**Keywords:** adrenal gland, diffuse large B-Cell lymphoma, ^18^F-FDG, PET/CT, SUVmax

## Abstract

**Rationale:**

Bilateral adrenal diffuse large B-cell lymphoma, nongerminal center B-cell phenotype (non-GCB DLBCL), is an uncommon malignancy that exhibits rapid development. Fluorine-18-fluorodeoxyglucose position emission tomography/computed tomography (CT) is extremely sensitive in distinguishing highly malignant tumors from benign tumors.

**Patient concerns:**

We report a case of non-GCB DLBCL showing significantly high uptake of 18F-FDG on PET/CT examination.

**Diagnoses:**

Histopathological and immunohistochemical examination further confirmed that the bilateral adrenal masses were non-GCB DLBCL.

**Interventions:**

The maximal standardized uptake value (SUVmax) of the adrenal lesion was 17.2. Abnormal 18F-FDG uptake was observed in a retroperitoneal lymph node, the SUVmax of which was 14.2.

**Outcomes:**

He was administered CHOP chemotherapy without rituximab due to high costs.His therapeutic effect and survival time could not be tracked due to patient privacy.

**Lessons:**

non-GCB DLBCL is a rare malignancy.18F-FDG PET/CT examination can distinguish benign from malignant adrenal lesions based on increased FDG uptake. It is a noninvasive method to diagnose malignant adrenal tumors.

## 1 Introduction

Primary adrenal lymphomas are extremely rare, and constitute 1% of non-Hodgkin lymphoma cases and 3% of extra nodal lymphomas; less than 200 cases have been known so far.^[1]^ It is bilaterally manifested in approximately 70% of cases.^[2]^ The major cases are diffuse large B-cell lymphomas, nongerminal center B-cell phenotype (non-GCB DLBCL).^[3]^ It is difficult to make a definite diagnosis based on symptom assessment. Treatment is a problem too. There is no standard treatment regimen for these tumors. They are always treated as non-Hodgkin lymphomas. The chemotherapy regimens are a standard treatment of non-Hodgkin lymphoma. The most common chemotherapy regimens used are R-CHOP (consisting of dexamethasone, cyclophosphamide, doxorubicin, and vincristine),^[4]^ and the prognosis of primary adrenal lymphoma is usually poor. There are some factors of poor prognosis, for example, advanced age, bilateral involvement, high lactate dehydrogenase levels, large tumor size, adrenal insufficiency at admission, nongerminal B-cell phenotype, metastasis to other organs, and bcl-6 rearrangement.^[3,5,6]^ Here, we describe a case of bilateral adrenal non-GCB DLBCL who showed significantly high uptake of ^18^F-FDG on PET/computed tomography (CT) examination. We hope that this examination can be helpful for early diagnosis of them and monitoring response to treatment and detecting recurrence.

## 2 Case presentation

### 2.1 Patient information

A 19-year-old male patient presented to our hospital on June 1, 2016, complaining of pain in the upper abdomen for 1 month, especially on the right side. After 1 month, he could not ignore the symptom. The patient had no previous history or family history of lymphoma.

### 2.2 Clinical findings

The vital signs of the patient were stable. When admitted to the hospital, his blood pressure was 100/61 mm Hg, pulse rate was 61 beats per minute, respiratory rate was 20 breaths per minute, and body temperature was 36.3 °C. Physical examination showed moderate anemia, pale palpebral conjunctiva, pharyngeal hematoma, abdominal tenderness, pain in the upper abdomen, and a slight knocking pain in the 2 kidneys.

### 2.3 Diagnostic assessment

Liver function tests revealed mild total hyperbilirubinemia (26.10 μmol/L), unconjugated hyperbilirubinemia (18.60 μmol/L), conjugated hyperbilirubinemia (7.50 μmol/L), and low levels of albumin (37.2 g/L). Liver enzymes were mildly out of normal range, with glutamic oxaloacetic transaminase levels of 50 IU/L. Renal function tests showed that the levels of uric acid (761 μmol/L) and beta 2 microglobulin (4.29 mg/L) were high.

**Figure 1:**
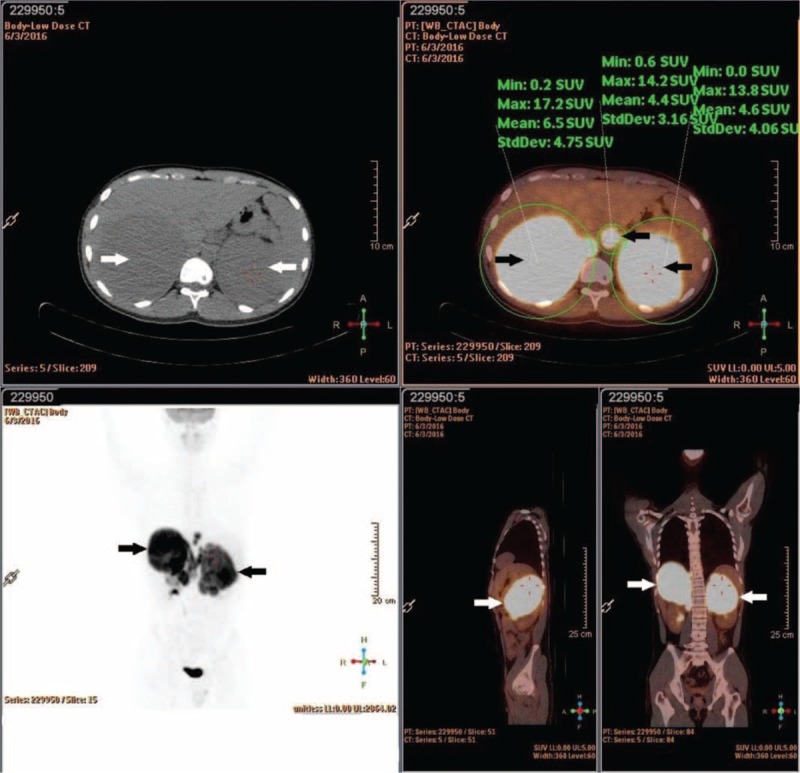
Abdominal CT showed bilateral adrenal masses (top left). ^18^F-fluorodeoxyglucose positron emission tomography (18-FDG/PET) scan (bottom left) and two fusion PET/CT images (top right and bottom right) showed increased FDG uptake in the bilateral adrenal glands and retroperitoneal lymph node. CT = computed tomography, 18-FDG = fluorine-18-fluorodeoxyglucose, PET = position emission tomography.

**Figure 2:**
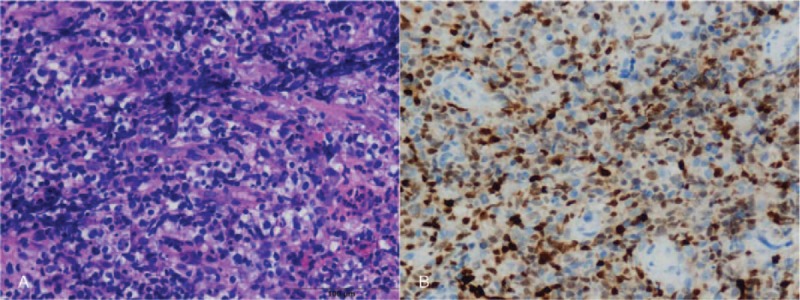
(A) Hematoxylin and eosin staining (×40) of tissue in left adrenal mass showed infiltration with large, irregular tumorous lymphocytes. (B) Immunohistochemistry with typic anti-MUM1 staining (×40) showed B lymphoid differentiation of these cells.

Blood routine examination revealed low red blood cell (1.80 × 10^12^/L), decreased levels of hemoglobin (64 g/L). Urinalysis, pretransfusion test, and routine stool examination results, and chest X-ray and electrolyte levels were within normal limits. CT images from the other hospital revealed a solid mass bilaterally located in the adrenal area. Subsequently, whole-body ^18^F-FDG PET/CT was performed to characterize the adrenal lesions and to identify the presence of metastases. ^18^F-FDG PET/CT (Fig. 1) showed significantly increased bilateral ^18^F-FDG uptake in the adrenal mass, the maximum size of which was 10.8×11.4 cm. The boundaries between the right mass and the liver, and those between the left mass and the left kidney were not clear. The maximal standardized uptake value (SUVmax) of the adrenal lesion was 17.2, which was suggestive of malignancy. There was also abnormal ^18^F-FDG uptake in a retroperitoneal lymph node; the SUVmax was 14.2, which was suggestive of metastasis (Fig. 1). Bone marrow biopsy showed that the hematopoietic tissues were proliferating actively, and the red blood cells were the most remarkable. A needle biopsy of the left mass confirmed non-GCB DLBCL. Hematoxylin and eosin staining (×40) of the left adrenal mass showed infiltration with large, irregular tumorous lymphocytes (Fig. 2A). The tumor cells expressed CD20, CD3 (partially), bcl-2, IRF4/MUM1, and bcl-6 (Fig. 2B), and they were negative for CD10, CD43, CD23, CD5, and cyclinD1 in immunohistochemistry.

### 2.4 Therapeutic intervention and outcomes

After diagnosis, he was also administered CHOP chemotherapy without rituximab due to high costs. Despite the administration of these regimes, his prognosis remained poor.

## 3 Discussion

Lymphomas are hematologic system tumors, and primary adrenal lymphomas are very rare. They are difficult to diagnose initially, as clinical symptoms are atypical, such as abdominal pain, fever of unknown origin, back pain, weight loss, anorexia, hypoglycemia, Addisonian crisis, and hyponatremia.^[7]^ Histopathological examination and immunohistochemistry are gold standards for diagnosis. The most common type is diffuse large B-cell lymphoma in both adrenal glands.^[2]^ Treatment includes surgery, radiotherapy, chemotherapy, and corticosteroid replacement therapy. The standard treatment regimen is R-CHOP chemotherapy in non-Hodgkin lymphoma.^[4]^ Primary adrenal lymphomas exhibit poor prognosis, although they present a positive initial reaction to drugs; remission when conditions improve is also rare after R-CHOP sschemotherapy.^[8]^ Especially in this patient, the prognosis must be poor due to bilateral involvement, large tumor size, non-germinal B-cell phenotype, metastasis to retroperitoneal lymph node, and bcl-6 rearrangement.

Although diagnosis is difficult, abdominal imaging is significantly helpful, especially ^18^F-FDG PET/CT. ^18^F-FDG PET/CT is reported to be highly sensitive in diagnosing lymphoma in recent years. It is based on higher glucose uptake at malignant sites and can distinguish between primary and secondary adrenal lymphomas. However, the distinguish between primary and secondary tumor is not absolutely accurate. We can combine the ^18^F-FDG PET/CT imaging of the whole body with related assisted examination to diagnose based on experience. A study by Cistaro et al^[9]^ revealed that ^18^F-FDG PET/CT offers better diagnostic performance than CT in the overall evaluation of patients with adrenal malignancies. In this case, the SUVmax of the adrenal mass was 17.2, which was suggestive of malignancy. Moreover, PET/CT revealed a retroperitoneal lymph node with SUVmax of 14.2, which was highly suggestive of metastasis. CT images only revealed solid masses located in the bilaterally adrenal area. It demonstrated non-GCB DLBCL based on histopathological findings and immunohistochemistry. Our result also showed that ^18^F-FDG PET/CT is better for diagnosis. Other studies show that ^18^F-FDG PET/CT examination has become a widely used imaging tool in diagnosing adrenal malignancies and has a great potential to characterize malignant lesions with high detection accuracy.^[10,11]^ In this case, the SUVmax of malignant lesions is high. High SUVmax seems to correlate with decreased survival and poor prognosis.^[12]^ But the study by Tessonnier et al^[13]^ showed that high SUVmax was not significantly associated with shorter overall survival and disease-free survival. So, additional studies are needed to further prove the relationship.

^18^F-FDG PET/CT cannot only be used for the diagnosis of bilateral adrenal non-GCB DLBCL, but also can be used to monitor response to treatment and detect recurrence.^[8]^ Unfortunately, the therapeutic effect and survival time could not be tracked due to patient privacy. We only know that the patient did not get better when he left.

In conclusion, regarding rare malignant adrenal tumors, this is a typical case with rich initial data. ^18^F-FDG PET/CT examination distinguishes benign from malignant adrenal lesions based on increased FDG uptake; this information is helpful for malignant adrenal tumors to be diagnosed using noninvasive methods.

## Author contributions

**Conceptualization:** Jing Zhou.

**Data curation:** Jing Zhou.

**Formal analysis:** Jing Zhou, Zhengxing Gou.

**Funding acquisition:** Jing Zhou.

**Investigation:** Jing Zhou, Yigang Zhao.

**Project administration:** Jing Zhou.

**Resources:** Jing Zhou.

**Validation:** Jing Zhou.

**Writing – original draft:** Jing Zhou.

**Writing – review & editing:** Jing Zhou.
